# Metastasis of gastrointestinal stromal tumor to skeletal muscle: a case report

**DOI:** 10.1186/1752-1947-8-256

**Published:** 2014-07-18

**Authors:** Kayo Suzuki, Taketoshi Yasuda, Kaoru Nagao, Takeshi Hori, Kenta Watanabe, Masahiko Kanamori, Tomoatsu Kimura

**Affiliations:** 1 Department of Orthopaedic Surgery, University of Toyama, 2630 Sugitani, Toyama, Toyama 930-0194, Japan; 2 Department of Orthopaedic Surgery, Iiyama Red Cross Hospital, Iiyama 226-1, Iiyama, Nagano 389-2295, Japan; 3 Department of Human Science 1, University of Toyama, 2630 Sugitani, Toyama, Toyama 930-0194, Japan; 4 Department of Orthopaedic Surgery, Faculty of Medicine, University of Toyama, 2630 Sugitani, Toyama, Toyama 930-0194, Japan

**Keywords:** Gastrointestinal stromal tumor, Sarcoma, Skeletal muscle metastasis

## Abstract

**Introduction:**

Gastrointestinal stromal tumor is the most common malignant mesenchymal tumor of the gastrointestinal tract. The most common sites of metastasis are the liver and the peritoneum, but gastrointestinal stromal tumors rarely metastasize to the skeletal muscles. Only three cases of gastrointestinal stromal tumor metastasizing to skeletal muscle have been reported in the English literature. Here we present an additional case of skeletal muscle metastasis, and the relevant literature is reviewed.

**Case presentation:**

A 54-year-old Japanese man presented with a three-month history of an enlarging mass of the left buttock. An excisional biopsy was performed and the tumor was diagnosed as a leiomyosarcoma. However, careful examination of the gastrointestinal tract revealed a tumor located in the small intestine. Surgical resection of the small intestine tumor was performed; histopathological and immunohistochemical examinations identified it as a primary gastrointestinal stromal tumor arising from the small intestine. Despite receiving both chemotherapy and molecular-targeted therapy, our patient died of gastrointestinal bleeding six months after the initial diagnosis.

**Conclusions:**

Because it is a mesenchymal tumor, it is difficult to distinguish a gastrointestinal stromal tumor metastasis to skeletal muscle from other primary soft tissue sarcomas. Although metastasis of gastrointestinal stromal tumor to skeletal muscle is rare, the likelihood of finding metastases in these unusual sites is increasing due to prolonged survival of patients with gastrointestinal stromal tumor after the introduction of imatinib therapy. We should include metastases of gastrointestinal stromal tumors as differential diagnosis of spindle cell tumor, and it is necessary to begin appropriate treatment early.

## Introduction

Gastrointestinal stromal tumors (GISTs) are the most common malignant mesenchymal tumors of the gastrointestinal (GI) tract, and account for 1 to 3% of all malignant GI tumors [1]. The precise cellular origin of GISTs recently has been proposed to be the intestinal cell of Cajal, an intestinal pacemaker cell. GISTs are most common in the stomach (60 to 70 percent), followed by the small intestine (25 to 35 percent), the colon and rectum (5 percent), and the esophagus (*<*2 percent). Based on a population-based sample from Southern Finland, Miettinen *et al.* originally estimated the incidence of malignant GIST to be four per million and the total incidence to be approximately 40 per million [2]. To the best of our knowledge, only three cases of GIST metastasizing to skeletal muscle have been reported in the English literature [3-5]. Here we present an additional case of skeletal muscle metastasis, and the relevant literature is reviewed.

## Case presentation

**Figure 1:**
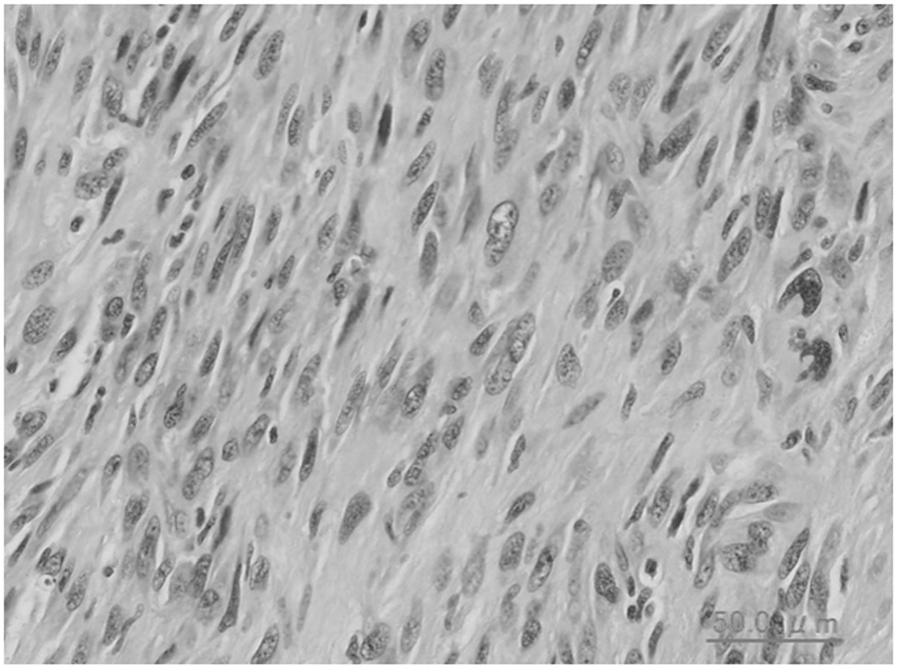
**Histopathological findings of the buttock mass at open biopsy.** Spindle-shaped tumor cells with atypical nuclei have proliferated. The initial diagnosis is a leiomyosarcoma (hematoxylin and eosin staining; scale bar, 50μm).

**Figure 2:**
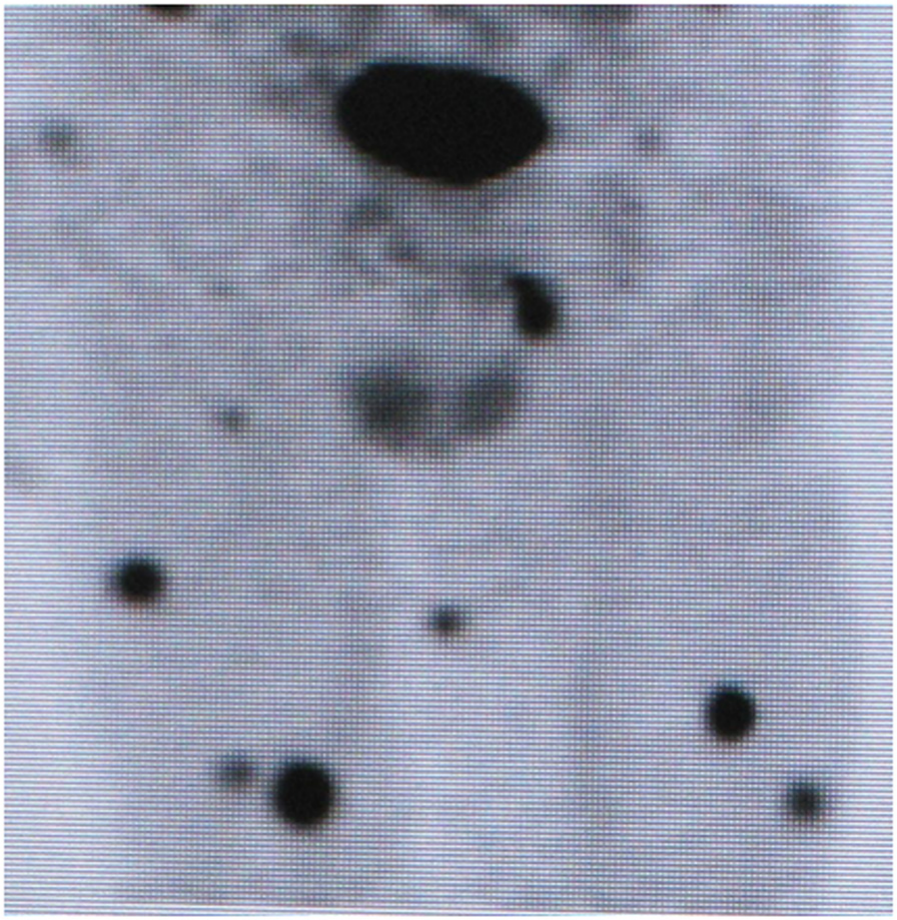
**Positron emission tomography-computed tomography scan.** Positron emission tomography-computed tomography scan shows multiple skeletal muscle metastases in the bilateral thighs.

**Figure 3:**
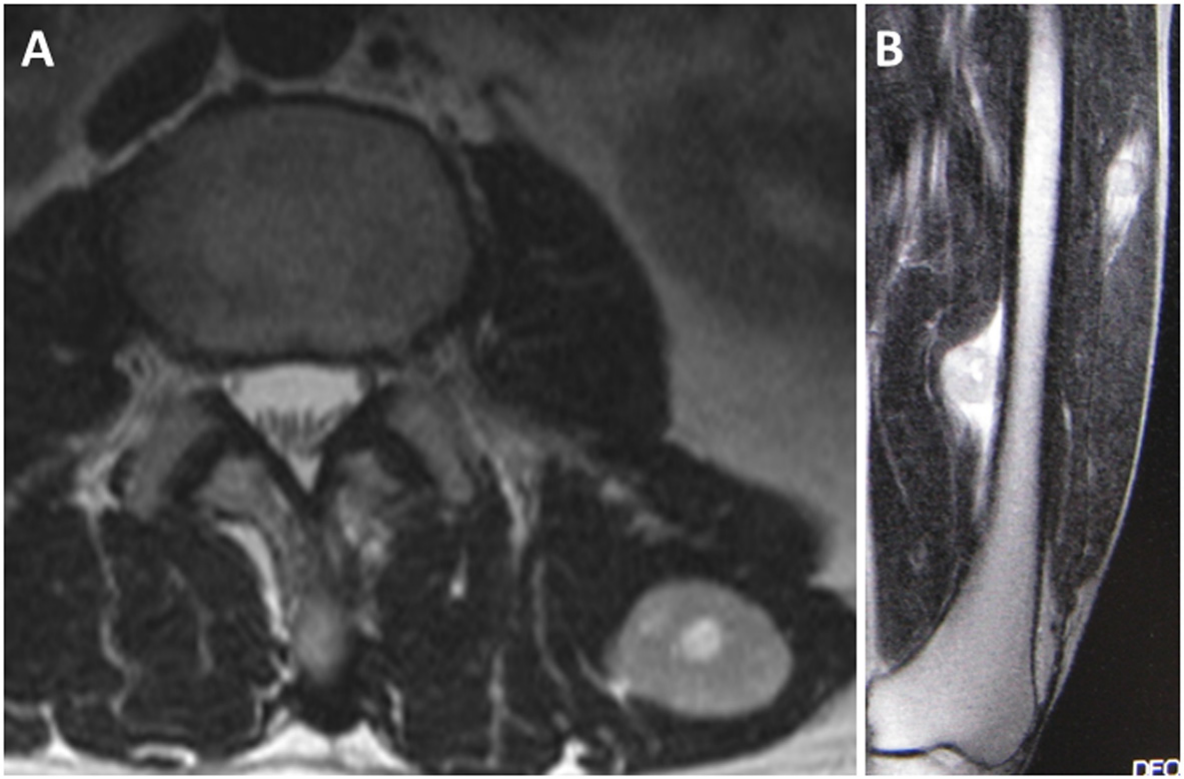
**Magnetic resonance imaging findings of the paravertebral muscle and left thigh.** Axial T2-weighted imaging reveals high-signal intensity masses in the paravertebral muscle **(A)** and the left quadriceps **(B)**.

**Figure 4:**
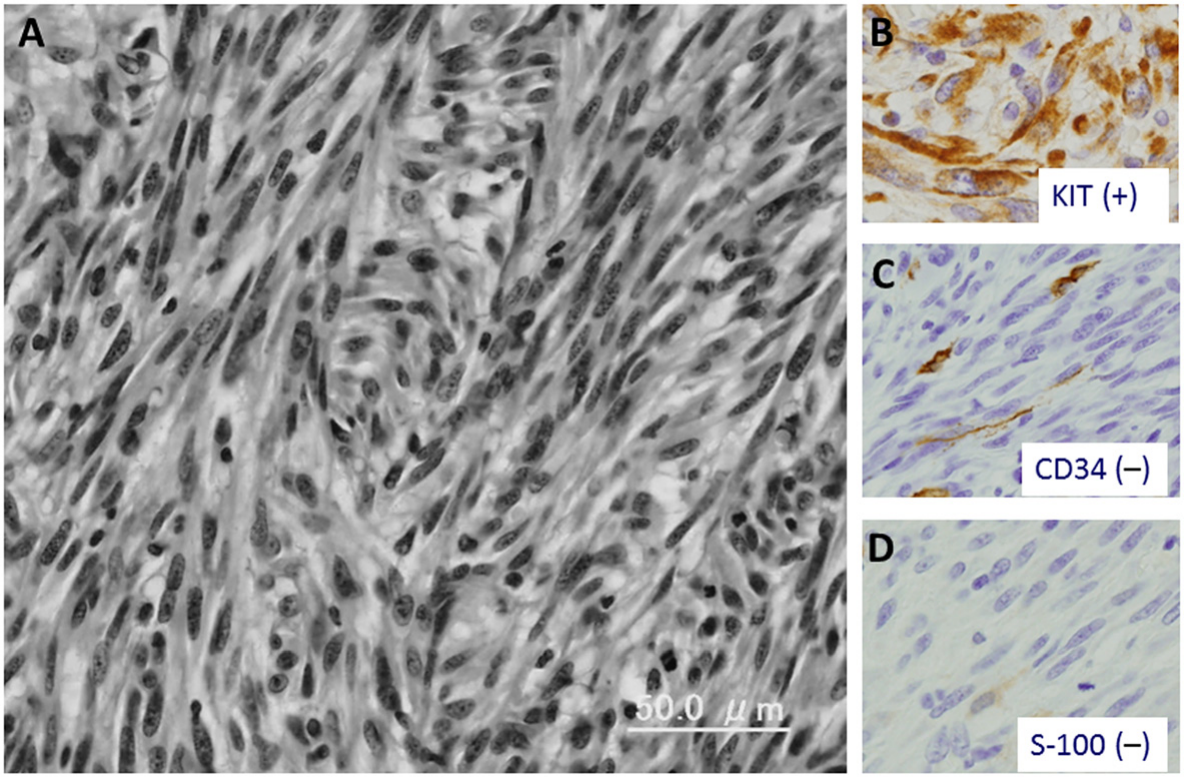
**Histopathological findings and immunohistochemical staining of the small intestine tumor. (A)** The resected mass shows a tumor with hypercellularity and spindle-shaped cells. The mitotic index is 70 per 50 high-power fields (hematoxylin and eosin stain, scale bar: 50μm). Immunohistochemical stain for c-KIT **(B)** shows diffuse staining of the cell membrane. CD34 **(C)** and S-100 **(D)** staining reveals negativity.

In 2010, three months prior to presentation, a 54-year-old Japanese man noticed an enlarging mass in the soft tissue of his left buttock. An excisional biopsy was performed and the tumor was diagnosed as a leiomyosarcoma composed of cellular bundles of spindle cells (Figure 1). Immunohistochemical stains demonstrated that tumor cells were positive for smooth muscle actin (SMA) and calponin, and negative for S-100, CD34 and epithelial membrane antigen (EMA). Subsequently, a positron emission tomography (PET)-computed tomography (CT) scan was performed. The results showed the existence of multiple metastatic lesions in the skeletal muscle and the absence of metastases in the liver, lung, and lymph nodes (Figure 2). The magnetic resonance (MR) images of the lumbar area and thigh revealed multiple isointense skeletal muscle tumors on T1-weighted with heterogeneous high-signal intensities on T2-weighted images (Figure 3A,B). Our patient was treated with doxorubicin (20g/m^2^ day 3), and ifosfamide (2,500g/m^2^ day 3) chemotherapy. Because he developed anemia during chemotherapy, a careful examination of his GI tract was performed, which revealed a bleeding tumor located in the small intestine. Surgical resection of the small intestine tumor was performed. Microscopically, the resected mass was composed of interlacing bundles of spindle and epithelioid mesenchymal cells with morphological features similar to the previously described tumors in his buttock. The mitotic index was 10/high-power field (HPF), and the tumor seemed to be a high-grade spindle cell sarcoma. Immunohistochemical analysis of the tumor cells revealed focal positivity for c-KIT and SMA, and negativity for CD34 and S-100 (Figure 4A-D). Based on these features, additional immunohistochemical analyses of the primary buttock tumor were performed. The buttock tumor cells were negative for c-KIT, but diffusely positive for platelet-derived growth factor receptor-α (PDGFRA) and were definitively diagnosed as a skeletal muscle metastasis of the primary small intestine GIST. Although our patient underwent chemotherapy with imatinib mesylate, death from GI bleeding occurred six months after the initial diagnosis.

## Discussion

GISTs are defined as pleomorphic mesenchymal tumors of the GI tract composed of spindle cells, epithelioid cells, or a combination of both, that express the c-KIT protein and, in most cases, CD34, as demonstrated by immunohistochemical staining. However, GISTs negative for c-KIT comprise 5 percent of all GISTs and contain a mutation of the *PDGFRA* gene that was reported to be related to the oncogenesis of these tumors [6]. Surgical resection of the local disease is the gold standard therapy. The goal is complete resection of the disease without tumor rupture. Tumor size, not negative microscopic surgical margins, determines survival. Complete surgical resection is possible in approximately 85 percent of patients, and 50 percent of patients will develop recurrence or metastasis following complete resection [7]. The five-year survival rate is approximately 50 percent, while the median time to recurrence after resection of primary high-risk GIST is two years. However, after the introduction of the molecular-targeted therapy, imatinib, there was a major improvement in the progression-free survival and overall survival rates. Nevertheless, GISTs have a high risk of metastatic relapse.

Skeletal muscle metastasis of carcinoma is relatively rare. Numerous case reports but few studies or large case series have been reported regarding this occurrence. Surov *et al.* reported a prevalence of 1.2 percent in 5,170 oncology patients. The frequency of skeletal muscle metastases varied from 0.4 to 4.9 percent for different primary tumors [8]. The most frequent occurrence of skeletal muscle metastasis was in patients with carcinoma of the cervix uteri, malignant melanoma, or ovarian carcinoma. Skeletal muscle metastases from sarcomas are extremely rare, as reported by Plaza *et al.*[9], who conducted the largest study concerning metastases to soft tissue; 118 patients with metastatic tumors to soft tissue were identified from a total of 7,237 soft tissue tumors and were included in their study (1.6 percent of all soft tissue sarcomas assessed during the same period). Of these 118 cases, 11 had metastases of sarcomatoid elements of primary soft tissue sarcomas from other sites, six had metastases from a uterine leiomyosarcoma, two from bone osteosarcoma, two from uterine malignant mixed Mullerian tumor, and one from ovarian carcinosarcoma. Arpaci *et al.* proposed to define the radiological imaging features and clinical findings of patients with skeletal muscle metastasis. In their study, the most common source was lung cancer and the muscles mostly affected by metastatic disease were the gluteals (15 percent), psoas (8.7 percent), paravertebral (8.7 percent), rectus abdominis (7.6 percent), and latissimus dorsi (6.5 percent) [10]. On the other hand, Bocchino *et al.* reported that most skeletal muscle metastases were localized in the psoas (33.3 percent), buttock (33.3 percent), and intercostal muscles (29.6 percent) [11]. Furthermore, Surov *et al.* showed that most muscle metastases were identified in the iliopsoas (27.5 percent), paravertebral (25 percent), gluteal (16.3 percent), lower extremity (12.5 percent), abdominal wall (10 percent), thoracic wall (5 percent), and upper extremity (3.8 percent) muscles [8].

To the best of our knowledge, this case is the fourth report of skeletal muscle metastases from a GIST (Table 1) [3-5]. The reports have included two cases of metastasis to muscle of the thigh, one to paravertebral muscle, and the current case, to the gluteal muscle. Although in two of the four cases the GIST had already metastasized to another site aside from skeletal muscle, two cases, including ours (cases one and four) were initially treated as leiomyosarcoma of the uterine or buttock muscle, respectively. Distinguishing between skeletal muscle metastases from GISTs and primary spindle cell sarcoma occurring in skeletal muscle may be challenging. Of a total of 133 rectal and anal GISTs identified in the Armed Forces Institute of Pathology at Washington and in the Haartman Institute of the University of Helsinki, 80 tumors (60 percent) had been originally diagnosed in other centers as leiomyosarcoma, 29 (21.8 percent) as smooth muscle tumors of uncertain malignant potential, 21 (15.8 percent) as leiomyoma and only three (2.25 percent) as GISTs [12]. Differentiating metastatic tumor from GIST and primary leiomyosarcoma in skeletal muscle has often been confusing. Our case was also, morphologically speaking, a spindle cell sarcoma with immunoreactivity for SMA.

**Table 1:** Clinical characteristics of GIST patients with skeletal muscle metastases

**Case**	**Reference [No.]**	**Age/Sex**	**Primary site**	Location of muscle metastases (years)^*^	**Initial DDx**	Other metastases (years)^*^	Outcome (years)^*^
1	Pasku *et al.*[3]	56/F	Pelvic cavity	Gluteal muscle (2)	Leiomyosarcoma	Lung (1)	NED (3)
2	Cichowitz *et al.*[4]	23/F	Small intestine	Adductor longus (over 5)	GIST	Liver (2)	AWD (over 5)
3	Bashir *et al.*[5]	56/M	Small intestine	Upper back muscle (within 1)	Leiomyosarcoma	Cardiac adrenal (1)	AWD (1)
4	Present case	54/M	Small intestine	Quadriceps (at the initial consultation)	Leiomyosarcoma	None	DOD (0.5)

^*^Period from the initial diagnosis. GIST, gastrointestinal stromal tumor; DDx, differential diagnosis; F, female; NED, no evidence of disease; AWD, alive with disease; M, male; DOD, died of disease.

Early recognition and prompt diagnosis will allow the proper treatment to be initiated in a timely fashion. Emmering *et al.* reported that PET-CT was a sensitive tool for early detection of skeletal muscle metastases and for staging malignant disease [13]. Bocchino *et al.* reported that 18-fluoro-fluorodeoxyglucose (^18^F-FDG) uptake in muscle metastatic lesions increased homogeneously reaching a higher maximum standardized uptake value (SUV_max_) (mean value 4.2 ± 2.8) than in the liver [11]. Surov *et al.* reported PET/CT findings of skeletal muscle metastases from different primary tumors [14]. Identified skeletal muscle metastases presented as intramuscular focal abdominal activity with SUVs ranging from 2.4 to 25.9, median SUV 7.8. The median size of the muscle metastases was 2.5cm (0.6 to 6.5cm). In this report, there were no significant correlations between SUV and size of muscle metastases or significant differences between SUVs of muscle metastases and primary tumors. Although MR imaging is not specific for skeletal muscle metastasis, it is an indispensable tool for diagnosis and treatment, the results of which clinicians may use to contemplate the general management of these patients. Surov *et al.* reported findings of MR imaging of intramuscular metastases [15]. On T2-weighted images, 97 percent of the recognized intramuscular metastases were hyperintense and on T1-weighted images, 91 percent of the intramuscular metastases were homogeneously isointense, in comparison to adjacent muscle tissue. The calculated apparent diffusion coefficient (ADC) values were low or moderate signal intensity in 94 percent of the intramuscular metastases.

In the current case, the findings of the MR imaging were hyperintensity on T2-weighted and isointensity on T1-weighted images in the identified location of the skeletal muscle metastasis, and according to the MR imaging results, our patient underwent excisional biopsy and the tumor was misdiagnosed as a leiomyosarcoma. We should remember to include metastases of GISTs in the differential diagnosis of spindle cell tumors, and that it is necessary to begin early and appropriate treatment.

## Conclusions

In summary, we present an extremely rare case of skeletal muscle metastasis from a GIST. To the best of our knowledge, there have been only three previously reported cases in the English literature of GIST metastasizing to skeletal muscle. Although skeletal muscle metastases from GIST are rare, the likelihood of finding metastases in these unusual sites has increased due to the prolonged survival of patients with GIST after the introduction of imatinib therapy. We should recognize metastases of GISTs in the differential diagnosis for spindle cell tumors, as it is necessary to begin appropriate treatment in a timely fashion.

## Consent

Written informed consent was obtained from the patient’s next-of-kin for publication of this case report and any accompanying images. A copy of the written consent is available for review by the Editor-in-Chief of this journal.

## Abbreviations

CT: computed tomography; EMA: epithelial membrane antigen; GI: gastrointestinal; GIST: gastrointestinal stromal tumor; HPF: high-power field; MR: magnetic resonance; PDGFRA: platelet-derived growth factor receptor-α; PET: positron emission tomography; SMA: smooth muscle actin; SUV: standardized uptake value.

## Competing interests

All authors have disclosed that they have no significant relationships with, or financial interest in, any commercial entity pertinent to this article.

## Authors’ contributions

KS was the major contributor in writing the manuscript. TY, KN, TH and KW supervised the treatment of our patient and collected case data. TY, MK and TK revised the manuscript. All authors approved the final version of the manuscript.
